# Palliative sedation at the end of life: Practical and ethical considerations

**DOI:** 10.1016/j.clinme.2025.100338

**Published:** 2025-06-14

**Authors:** Caroline Barry, Robert Brodrick, Gurpreet Gupta, Imranali Panjwani

**Affiliations:** a Norwich Medical School, University of East Anglia, Norwich; b Department of Palliative Care Medicine, Norfolk and Norwich University Hospital NHS Trust, Norwich; c Clinical School, University of Cambridge, Cambridge; d St Luke’s Hospice, Harrow and London North West University Healthcare NHS Trust, Harrow; e Faculty of Business & Law, Anglia Ruskin University, Cambridge

**Keywords:** Palliative, End-of-life care, Sedation, Ethics, Consent

## Abstract

Sedation is commonly used at the end of life; however, there are several practical and ethical considerations for its use. It is important to identify any treatable causes for agitation prior to initiating medication. The drug, dose and route of administration may vary according to the indication for treatment, and specialist advice or supervision may be required. There are a number of ethical and cultural considerations relevant to the use of palliative sedation, which must also be understood to ensure best practice in this area.

## Highlights

• The aim of palliative sedation is to relieve refractory suffering with the use of medications to reduce consciousness.
• Where palliative sedation is being used to treat agitation at the end of life, it is important to exclude and/or address reversible causes prior to starting medication.
• The drug, dose and route of administration of palliative sedation may vary according to the indication for treatment.
• Appropriate and proportionate use of palliative sedation does not hasten death.
• Suffering may have different meanings for people depending on their backgrounds and life experiences. These should be explored prior to starting palliative sedation.

## Introduction

The aim of palliative sedation is *‘to relieve refractory suffering through the monitored, proportionate use of medications intended to reduce consciousness in patients with life-limiting disease.’*^1^

In this paper we describe the clinical practice of palliative sedation, explore what is meant by ‘refractory suffering’ in this context, and consider circumstances in which it may, or may not, be considered ethically acceptable to do so.

## Principles of palliative sedation

The suffering associated with life-limiting disease can be severe and challenging to alleviate. When other options for relieving such distress have been exhausted, it is common practice for doctors to intentionally reduce a patient’s level of consciousness. The prevalence of palliative sedation described in the literature ranges from 12 to 67%,^2^^,^^3^ and midazolam is one of the three most administered drugs in palliative care.^4^

There is considerable variation in decision making for palliative sedation.^5^ There is little evidence based on high-quality, prospective data.^6^ Before starting palliative sedation, clinicians must be confident and adept in their assessment of refractory symptoms, and the ability to differentiate between somatic, psychological and existential suffering.^1^

## Indications

Palliative sedation may be used in the following circumstances:^1^

1. For the management of refractory suffering.
2. In emergency situations where death is both imminent and anticipated to be distressing without sedation.
3. Withdrawal of life-sustaining interventions where symptoms are likely.
4. As temporary respite when other symptom-focused treatment cannot achieve sufficient relief in an acceptable time frame.

Common examples are provided in Table 1.

**Table 1:** Indications for palliative sedation.

Symptom control	• Terminal agitation • Management of terminal haemorrhage • Management of irreversible airway obstruction • Patient request for continuous deep sedation (specialist use only) • Adjunctive treatment of intractable pain (specialist use only)
Withdrawal of treatment	• Withdrawal of ventilatory support • Withdrawal of clinically assisted hydration and nutrition

**Fig. 1:**
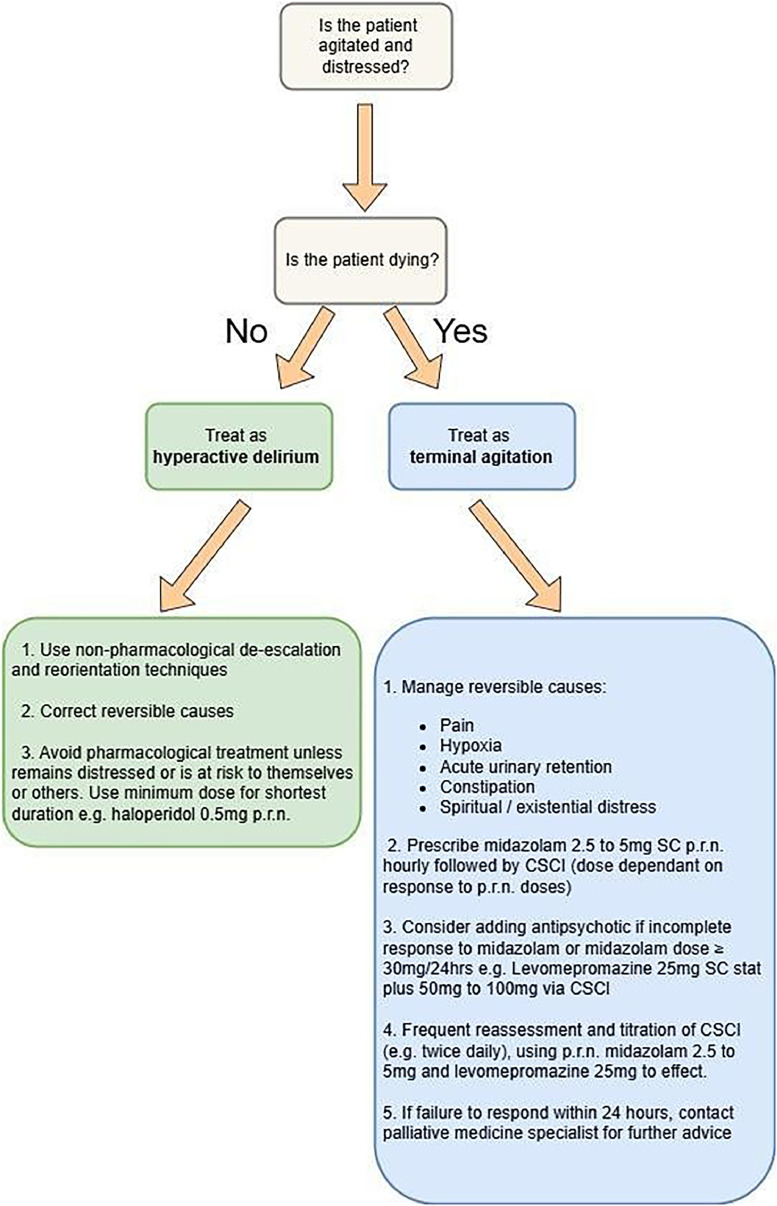
Treatment of terminal agitation.

The most common use for sedation in palliative care is for terminal agitation (sometimes referred to as terminal restlessness or agitation in the imminently dying). It is characterised by distress, anxiety and restlessness in the final days of life. In contrast to standard delirium treatment, pharmacological measures are often considered first line as shown in Fig. 1, after the exclusion of reversible causes.

Where the possibility of major haemorrhage is foreseen, the patient is normally counselled regarding this possibility. An ‘anticipatory’ bolus dose of sedative medication may be prescribed to achieve rapid unconsciousness (see Table 2). In cases of catastrophic arterial bleeding, where death is anticipated within seconds or minutes, there is usually not sufficient time to achieve palliative sedation. Priority should instead be given to remaining calm and staying with the patient.

**Table 2:** Commonly used medication, doses and route of administration.

Indication	Medications	Typical doses	Notes
Terminal agitation^7^	Midazolam	2.5–5 mg SC hourly p.r.n. 10–100 mg/24 h via CSCI	Often used first line. Used at lower doses to reduce anxiety or cognitive agitation.
	Levomepromazine	12.5–25 mg SC hourly p.r.n. 25–200 mg/24 h via CSCI	Used when reduction of consciousness is intended. Causes postural hypotension. Lowers seizure threshold. Used at lower doses to manage nausea/vomiting.
	Haloperidol	1.5–2.5 mg SC hourly SC p.r.n. 2.5–10 mg/24 h via CSCI	Used when either distressing hallucinations or psychomotor agitation is present. Less sedating than levomepromazine but higher risk of extrapyramidal side effects.
	Phenobarbital	200 mg IV or IM loading dose and then hourly p.r.n. 800–1,600 mg/24 h via CSCI	Avoid SC p.r.n. use due to risk of tissue necrosis. Specialist use only.
Major haemorrhage and irreversible airway obstruction^7^	Midazolam	5–10 mg buccal/IV/IM/PR p.r.n. every 30 min	Priority should be to stay calm and present with the patient. The subcutaneous route should be avoided in haemorrhage due to risk of reduced perfusion and delayed absorption.
NIV withdrawal^8^	Midazolam	2.5–5 mg IV boluses or 5–10 mg SC boluses Repeated every 5 min (IV) or 20 min (SC) until unconscious	Usually co-prescribed with an SC/IV opioid. CSCI started if survival is prolonged. Seek specialist guidance.
Elective withdrawal of clinically assisted hydration and nutrition^9^	First line: midazolam	10–100 mg/24 h via CSCI	Only under specialist supervision following established clinical guidelines.
	Second line levomepromazine	25–200 mg/24 h via CSCI	
Patient request for continuous deep sedation	Midazolam	2.5–5 mg SC hourly p.r.n. 10–100 mg/24 h via CSCI	Ethically and legally contentious, depending on the specific context. Specialist use only.
	Levomepromazine	12.5–25 mg SC hourly p.r.n. 25–200 mg/24 h via CSCI	
	Phenobarbital	200 mg IV or IM loading dose and then hourly p.r.n. 800–1,600 mg/24 h via CSCI	

SC, subcutaneous; IV, intravenous; IM, intramuscular; CSCI, continuous subcutaneous infusion; NIV, non-invasive ventilation.

Palliative sedation for the withdrawal of life-sustaining treatment must be considered in the context of a wider clinical plan. This normally requires multidisciplinary team (MDT) decision making, meticulous documentation and discussion with those important to the patient. Consensus national guidance exists to support clinicians in specific scenarios, such as the withdrawal of non-invasive ventilation and of clinically assisted hydration and nutrition.^8^^,^^9^

It should be explained, to both the patient and those important to them, that palliative sedation is used to address refractory and intolerable suffering, not hasten death. There is no evidence that the appropriate use of sedation shortens prognosis in any context.^2^

## Key management steps

### Medications

#### Benzodiazepines

Midazolam is the benzodiazepine of choice for palliative sedation due to its high parenteral bioavailability, widespread compatibility with other commonly used palliative medications, and low cost. Lower doses (5–10 mg/24 h via CSCI) are predominantly anxiolytic and are often consciousness sparing. Midazolam is used first line for patients with anxiety (where the oral route is not appropriate) and cognitive agitation. Higher doses (up to 100 mg/24 h via CSCI) cause progressively deeper levels of sedation where indicated (Table 2).

#### Antipsychotics

Levomepromazine and haloperidol are used in palliative care for both terminal agitation and the management of nausea and vomiting. Higher doses are required when used for sedation. Antipsychotics are often used first line when patients experience psychomotor agitation or distressing hallucinations. The choice of antipsychotic may be influenced by the likelihood of additional beneficial or adverse effects. Levomepromazine is more sedating than haloperidol and works synergistically with midazolam. In the UK, it is the preferred antipsychotic for severe terminal agitation. It is added when midazolam alone is insufficiently sedating and a reduction of conscious level is intended. Internationally, some centres use olanzapine or chlorpromazine as alternatives.

#### Barbiturates

Phenobarbital can be used when antipsychotic medication is either relatively contra-indicated (eg Parkinson’s disease) and/or in the presence of agitation refractory to high-dose midazolam and levomepromazine. As higher doses are associated with respiratory depression, it should only be initiated under specialist supervision.

#### Other medications

Propofol is a short-acting, intravenous anaesthetic conventionally used for the induction and maintenance of anaesthesia, or for sedation in intensive care. Use of propofol is rare in UK palliative care practice, but is more common in continental Europe.^1^ Administration under the supervision of an anaesthetist is strongly advisable. The alpha_2_-adrenoreceptor agonist dexmedetomidine may be effective at reducing terminal agitation with less sedation than traditional sedatives.^10^, ^11^

#### Indications for palliative sedation: medications, doses and route

Deeper levels of sedation are associated with loss of the oral route. Subcutaneous injection, intravenous injection, rectal and buccal routes produce rapid effects of relatively short duration. When prolonged sedation is required, continuous subcutaneous infusion (CSCI) provides consistent medication administration over 24 h regardless of conscious level. When the patient has already developed symptoms of terminal agitation, it is appropriate to give a bolus subcutaneous injection, before starting a CSCI which may take 3–4 hours to reach full effect.

### The nature of suffering: cultural considerations

A patient’s relationship to their suffering must be considered before deliberately reducing their conscious experience.

Suffering can be described as the *a posteriori* struggle that a person goes through in their life when met with physical, psychological, spiritual and/or social challenges.^12^ If a person believes that their suffering has an *a priori* purpose, it is often experienced as more bearable, and may even be perceived as valuable.^13^

The purpose of suffering is a major theme in many religious and philosophical traditions. For example, the Qur’an, Torah, Bible, Bhagavad Gita and Pali Canon all emphasise that suffering in life is temporary. Such suffering is often contrasted with an enduring state of ease or bliss after death as well as self-realisation.^14^ Palliative sedation, even for severe suffering at the end of life, may be undesirable for some patients; for example, if they believe that it is not congruent with their values or that it may jeopardise their experience of an afterlife.

Ultimately, that which seems purposeless to clinicians may hold profound significance to patients. If a patient’s values and beliefs are not explored, the prospect of palliative sedation may increase their suffering, rather than reduce it.

### Ethical considerations

For palliative sedation to be practised ethically, prescribers must understand its legitimate indications, justifications and aims.

Historically, there has been wide variability in terminology and sedation practice.^1^ For example, a recent European-wide systematic review of palliative sedation practice found that most studies did not define ‘refractory’.^15^ There is currently no standard tool for refractory symptom assessment.

When palliative sedation is considered for the management of terminal agitation, the dying person will commonly have impaired capacity to consent to treatment. The best interests of the individual should therefore be the guiding ethico-legal principle. Ambiguity as to the aims and justifications of such treatment may lead to medication administration based on other reasons, for example to relieve carer distress.

Mistrust of clinicians may complicate discussions around palliative sedation. For example, individuals from minoritised communities may doubt the compassionate intentions of clinicians offering palliative sedation. Use of restrictive practices such as sedation, restraint and detention of people of Black, Asian and minority ethnic backgrounds in other medical contexts have been known to be disproportionate and unethical.^16^, ^17^, ^18^ Acknowledging this is crucial to ensuring transparency and maintaining trust.

When sedation is prescribed for patients who are not imminently dying, it is important that the level and duration of sedation does not compromise their nutrition and hydration status. Either the level of consciousness achieved must permit sufficient oral nutrition and hydration to sustain life, or arrangements must be put in place to provide clinically assisted hydration and nutrition. In the UK, deep sedation of a patient with a prognosis of greater than 1–2 weeks without maintenance of nutrition and hydration is both poor practice and legally problematic. Such an approach, sometimes termed ‘slow euthanasia’, undermines public trust in the legitimate use of palliative sedation.^19^

## Conclusion

The use of sedation with the aim of reducing suffering in palliative care is widespread. Indications and practices vary between settings, although clinical guidelines and formularies exist to support practice. Palliative sedation poses several practical and ethical challenges. Clinicians should aim to provide an individualised approach to patient assessment, working closely with specialist palliative care colleagues and the wider MDT.

## Funding

No external funding was received for this paper. CB is supported by the East of England Applied Research Collaboration (ARC).

## CRediT authorship contribution statement

**Caroline Barry:** Writing – review & editing, Writing – original draft, Conceptualization. **Robert Brodrick:** Writing – review & editing, Writing – original draft, Conceptualization. **Gurpreet Gupta:** Writing – review & editing, Writing – original draft. **Imranali Panjwani:** Writing – review & editing, Writing – original draft.

## Declaration of competing interest

The authors declare that they have no known competing financial interests or personal relationships that could have appeared to influence the work reported in this paper.
